# Supplementary material for: Towards a taxonomically unbiased European Union biodiversity strategy for 2030
Source: Proc Biol Sci. 2020 Dec 9;287(1940):20202166. doi: 10.1098/rspb.2020.2166 (PMC7739930; doi:10.1098/rspb.2020.2166)
Supplement: Supplementary Material – combined document with more that 5 files [file rspb20202166supp1.pdf]

**Supplementay materials**

*Proceedings of the Royal Society B: Biological Sciences*

doi: 10.1098/rspb.2020.2166

# Supplementary material for:

# Towards a taxonomically unbiased European Union Biodiversity Strategy for 2030

Stefano Mammola, Nicoletta Riccardi, Vincent Prié, Ricardo Correia, Pedro Cardoso, Manuel Lopes-Lima, Ronaldo Sousa

## Supplementary items:

**Table S1:** LIFE projects investment breakdown among animal groups

**Table S2:** Estimated regression parameters for the negative binomial Generalized linear mixed model

**Figure S1**: Multicollinearity among continuous variables.

**Figure S2**: Association between continuous and categorical variables.

**Figure S3**: Relationship between body size and species' public interest.

doi: 10.1098/rspb.2020.2166

**Table S1:** LIFE projects investment breakdown among animal groups (1992–2018; n = 835 LIFE projects).

| **Animal group** | **Number of species** | **Total funding [Euro]** |
|---|---|---|
| Amphibian | 34 | 53,424,690.98 |
| Birds | 216 | 447,771,421.63 |
| Fish | 62 | 135,258,976.43 |
| Mammals | 76 | 387,716,614.01 |
| Reptiles | 21 | 46,103,756.32 |
| Invertebrates | 77 | 154,577,014.64 |

doi: 10.1098/rspb.2020.2166

**Table S2:** Estimated regression parameters for the negative binomial Generalized linear mixed model. S.E. = standard error; EX: Extinct; CR: Critically Endangered; EN: Endangered; VU: Vulnerable: NT: Near Threatened; LC: Least Concern; DD: Data Deficient.

| **Variable** | **Estimated β ± S.E.** | ***p-value*** |
|---|---|---|
| Intercept (IUCN baseline: CR) | 0.53 ± 033 | - |
| IUCN – EX | 0.05 ± 1.03 | 0.95 |
| IUCN – EN | 0.36 ± 0.33 | 0.27 |
| IUCN – VU | –0.03 ± 0.31 | 0.90 |
| IUCN – NT | 0.53 ± 0.31 | 0.07 |
| IUCN – LC | 0.17 ± 0.28 | 0.55 |
| People interest (logarithm) | 0.18 ± 0.03 | < 0.001 |
| Body size (logarithm) | –0.07 ± 0.05 | 0.17 |

doi: 10.1098/rspb.2020.2166

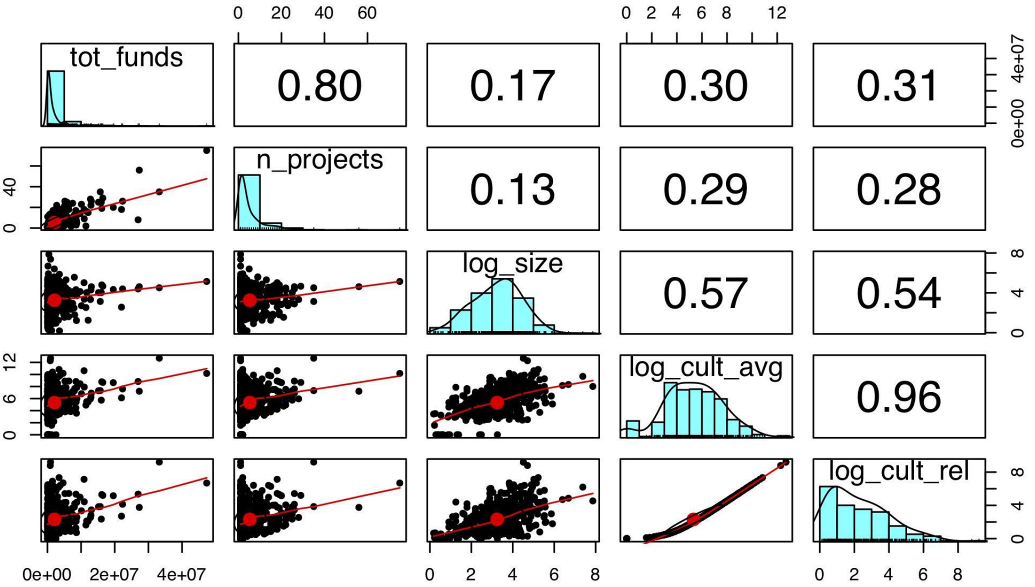


**Figure S1:** Multicollinearity among continuous variables. Above the diagonal: Pearson *r* correlation coefficient. On the diagonal: histogram illustrating data distribution. Above the diagonal: scatter plot of x by y with a trendline fitted through the data. tot_funds: total monetary investment; n_projects: N° of LIFE projects; log_size: body size (logarithm); log_cult_avg: average people interest (logarithm); log_cult_rel: relative people interest (logarithm).

doi: 10.1098/rspb.2020.2166

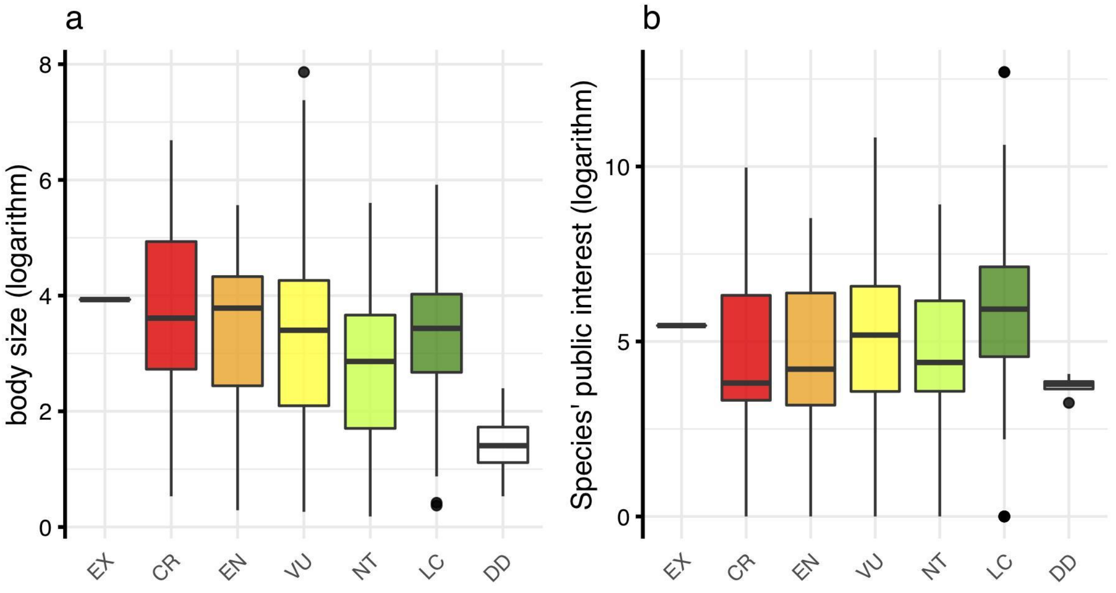


**Figure S2:** Association between continuous and categorical variables used in the regression models. **a**) Body size **b**) Species' online popularity.

doi: 10.1098/rspb.2020.2166

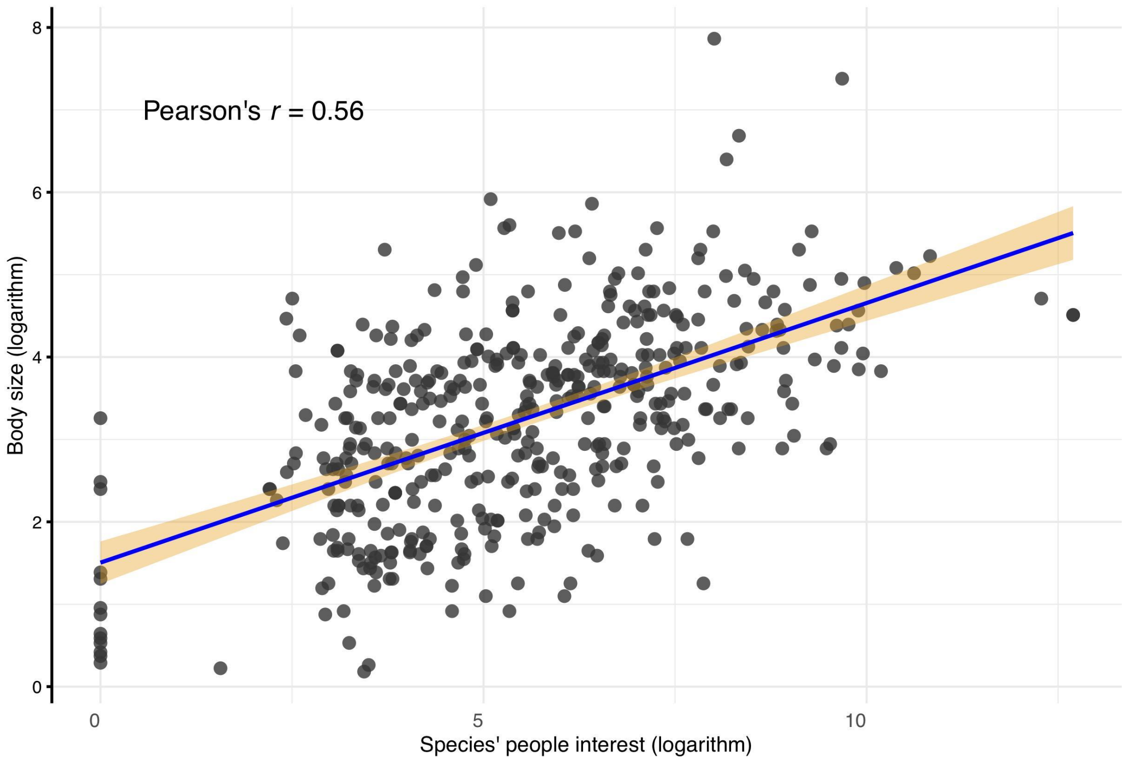


**Figure S3:** Relationship between body size and species' online popularity.
